# Effect of PPC Content on the Structure and Properties of PBAT/PLA/PPC Ternary Composite Mulch Films

**DOI:** 10.3390/polym18182228

**Published:** 2026-09-12

**Authors:** Rui Xu, Zhiyu Zheng, Zhichao Lou, Lei Xu

**Affiliations:** 1 Institute of Agricultural Facilities and Equipment, Jiangsu Academy of Agricultural Sciences, Key Laboratory for Protected Agricultural Engineering in the Middle and Lower Reaches of Yangtze River, Ministry of Agriculture and Rural Affairs, Nanjing 210014, China; 20210080@jaas.ac.cn (R.X.); 20240025@jaas.ac.cn (Z.Z.); 2 National-Provincial Joint Engineering Research Center of Biomaterials for Machinery Package, Nanjing Forestry University, Nanjing 210037, China

**Keywords:** poly(propylene carbonate), poly(butylene adipate-co-terephthalate), poly(lactic acid), biodegradable mulch films, critical compatibility threshold

## Abstract

The binary blend of poly(butylene adipate-co-terephthalate) (PBAT) and poly(lactic acid) (PLA) has emerged as the primary base material combination for biodegradable mulch films because of their favorable processability and biodegradability, offering a viable route to replace conventional polyethylene films in agricultural applications. However, the PBAT/PLA binary system suffers from thermodynamic incompatibility, limiting simultaneous achievement of mechanical, barrier, and optical properties. This study introduces poly(propylene carbonate) (PPC) as the third component and investigates its content (0–20%) on the microstructure and performance of PBAT/PLA/PPC ternary films. At PPC ≤ 10%, the system maintains an amorphous homogeneous structure, and PPC enriches the surface and improves interfacial adhesion. When the PPC content is 10%, the blend exhibited a transverse tensile strength of 43.3 MPa, an elongation at break of 338%, and a 29.8% reduction in water vapor permeability versus the neat blend. At 15% PPC, the compatibility threshold is exceeded, causing severe phase separation, a sharp drop in melt strength, and deteriorated mechanics. At 20%, phase separation induces PBAT/PLA crystallization, further enhancing barrier performance but reducing thermal stability and transparency. Differential scanning calorimetry and dynamic rheological analysis confirm the compatibility threshold, while X-ray photoelectron spectroscopy, X-ray diffraction, and scanning electron microscopy reveal abrupt changes in surface chemistry, crystal structure, and morphology. Overall, 10% PPC offers the best balanced properties. This work elucidates structure and property relationships, providing a basis for the rational formulation design of biodegradable mulch film. Further field weathering and biodegradation tests are required to validate their practical agricultural performance.

## 1. Introduction

Plastic mulch cultivation is an essential practice in modern agriculture. By conserving heat and soil moisture and suppressing weed growth, plastic mulching can substantially improve crop yields and resource-use efficiency [1,2,3]. China is the world’s largest producer and user of agricultural plastic mulch, with an annual coverage area of nearly 300 million mu and annual consumption accounting for approximately 75% of the global total [4,5]. However, the long-standing emphasis on the use rather than the recovery of conventional polyethylene (PE) mulch has resulted in the accumulation of approximately 2.07 million tonnes of residual plastic film in China’s agricultural soils [6,7]. Such residues reduce soil permeability [8,9], disrupt microbial communities [10], and impede crop root development [11], posing a serious threat to the health and functioning of farmland ecosystems [12]. To address agricultural “white pollution” and protect soil health, biodegradable mulch films are increasingly being used because they can degrade in situ and eliminate the need for post-use collection [13,14] as alternatives to conventional polyolefin films [15,16,17]. In recent years, the Chinese government has continuously strengthened its policy support to promote the research, development, and application of biodegradable mulch films, aiming to address agricultural plastic film pollution while maintaining crop productivity. This policy-driven push has accelerated the transition from traditional PE films to biodegradable alternatives, making the development of high-performance, cost-effective biodegradable mulch films an urgent practical need.

Among the various degradable polymers available, binary blends of poly(butylene adipate-co-terephthalate) (PBAT) and polylactic acid (PLA) have become widely used base materials for commercial biodegradable mulch films because of their favorable processability and biodegradability [18]. PBAT contains both aliphatic and aromatic segments in its molecular chains, which give the material excellent flexibility and film-forming properties [19]. PLA, by contrast, is known for its high tensile strength and excellent transparency [20]. Their complementary properties therefore make PBAT and PLA, in principle, a promising combination for mulch-film applications [21]. Nevertheless, numerous studies have shown that PBAT and PLA are thermodynamically incompatible. PLA has relatively rigid molecular chains with methyl-containing side groups, whereas PBAT consists of both aliphatic and aromatic segments. During melt blending, differences in molecular polarity and mismatched thermodynamic parameters tend to produce a sea–island phase-separated morphology with weak interfacial adhesion between the two phases [22]. In terms of mechanical properties, this phase separation results in an elongation at break considerably lower than the theoretically additive value, thereby limiting the toughening effect [23]. It also compromises the water vapor barrier performance. The numerous microscopic voids and defects at the interface between the two phases provide pathways for water-molecule permeation, making it difficult to effectively reduce the water vapor transmission rate of PBAT/PLA binary films and consequently weakening their ability to retain soil moisture [24]. In addition, the PBAT/PLA binary system has limited UV-shielding capability and offers relatively little scope for tuning optical properties. These limitations make it difficult for the material to simultaneously meet the requirements for soil warming, moisture conservation, and weather resistance under actual field conditions [25].

Introducing a third component to form a ternary blend provides a viable approach to overcoming these limitations and improving the overall performance of the PBAT/PLA matrix. Considerable research has focused on improving interfacial compatibility between the PBAT and PLA phases by incorporating fillers such as montmorillonite [26], calcium carbonate [27], lignin [28], and starch, or by using organic compatibilizers such as epoxy chain extenders [29] and isocyanate-based reactive additives [30]. These approaches, however, may cause irreversible losses in film transparency [31] or compromise the environmental compatibility of the material through the introduction of non-degradable components [32,33]. In comparison, physical blending with a third polymer that is itself fully biodegradable is more consistent with the requirements for degradability and sustainable use of agricultural mulch films. Polypropylene carbonate (PPC) is an aliphatic polycarbonate synthesized through the copolymerization of carbon dioxide and propylene oxide. Its molecular chains contain abundant carbonate groups and ether linkages. PPC can exhibit an elongation at break exceeding 600%, has very low water vapor permeability, and possesses intrinsic UV-absorbing properties [34]. More importantly, the carbonate groups and terminal hydroxyl groups along the PPC chains can form intermolecular interactions with the carboxyl and hydroxyl groups present in the PBAT/PLA phases. These interactions can strengthen interfacial adhesion and reduce microscopic voids at the phase boundaries, thereby improving both the barrier properties and toughness of the blend [35]. PPC is therefore widely regarded as a multifunctional modifier capable of simultaneously providing toughening, compatibilization, and enhanced barrier performance.

A series of exploratory studies on PBAT/PLA/PPC ternary blends have been conducted in China and elsewhere. Wang et al. [36] prepared PBAT/PPC/PLA ternary mulch films by melt blending followed by blown-film processing. At a PBAT/PPC/PLA ratio of 64/20/16 and a blow-up ratio of 3.1, the films achieved a longitudinal tensile strength of 43.0 MPa and a transverse elongation at break of 450%. Dynamic mechanical analysis and differential scanning calorimetry further indicated partial compatibility among the three components. With the subsequent addition of a highly efficient hindered amine light stabilizer, the films retained an elongation at break above 100% after 100 h of UV aging, effectively extending their service life. Choo et al. [37] systematically investigated the reactive compatibilization of epoxidized soybean oil (ESO) in PLA/PBAT/PPC ternary blends. They found that the epoxy groups of ESO underwent ring-opening reactions with the terminal carboxyl and hydroxyl groups of PLA, PBAT, and PPC, leading to the formation of branched structures. At an ESO content of 4 phr, the elongation at break increased markedly from 12.75% to 49.65%, representing an increase of 389%. However, the plasticizing effect of ESO increased the free volume of the blend and consequently reduced its oxygen barrier performance. Guo et al. [38] used PBAT as the matrix and incorporated PLA and PPC through melt blending with a PTLA compatibilizer. Compared with neat PBAT films, the resulting modified composite mulch films showed a 58.62% increase in tensile strength and a 70.33% improvement in water vapor barrier performance, while their functional service period was extended by 30 days. Field experiments with maize further showed that the improved heat and moisture retention provided by the films increased maize yield by 5.45% relative to the PBAT-film treatment, bringing it close to the level achieved with conventional PE mulch. Although these studies have demonstrated the potential of PBAT/PLA/PPC blends, their studies were primarily limited to selected formulations or focused on specific properties such as aging or reactive compatibilization. To date, a systematic investigation on how the PPC content continuously influences the microstructural evolution, such as surface chemistry, crystalline structure, phase morphology, and its consequent impact on the comprehensive performance of the ternary system is still lacking. In particular, the existence of a critical compatibility threshold and its decisive role in determining the mechanical and barrier properties have not been revealed.

In this study, PBAT/PLA at a ratio of 90/10 was used as the base formulation, and a series of ternary biodegradable composite mulch films were prepared with PPC at mass fractions of 0%, 5%, 10%, 15%, and 20%. First, melt strength measurements, scanning electron microscopy, and differential scanning calorimetry were used to examine how PPC content affects the melt-processing characteristics of the ternary composite pellets and the compatibility among the constituent polymers. X-ray diffraction and X-ray photoelectron spectroscopy were then employed to further investigate the effects of PPC content on the chemical bonding state, functional-group interactions, and crystalline structure of the composite films. Mechanical testing with a universal testing machine, thermogravimetric analysis, and measurements of the water vapor transmission coefficient, UV transmittance, and light transmittance were subsequently performed to systematically evaluate the effects of PPC content on the mechanical properties, thermal stability, water vapor barrier properties, UV-shielding performance, and light transmittance of the composite films. On this basis, the structure–property relationships among PPC content, microstructure, and macroscopic properties were elucidated. This study aims to provide a theoretical basis and supporting data for the compositional design of high-performance biodegradable mulch films based on the PBAT/PLA/PPC system.

## 2. Materials and Methods

### 2.1. Materials

Poly(butylene adipate-co-terephthalate) (PBAT, C1200, BASF SE, Ludwigshafen am Rhein, Germany) exhibited a density of 1.25 g·cm^−3^, a melt flow rate (MFR) of 3.7 g·10 min^−1^ (190 °C, 2.16 kg; ISO 1133-1:2022 [39]), and a melting temperature of 110–120 °C. Polylactic acid (PLA, 4032D, NatureWorks LLC, Plymouth, MN, USA) exhibited a density of 1.24 g·cm^−3^ and molecular weight characteristics of Mn = 9.4–10.6 × 10^4^ g/mol, Mw = 1.68–2.14 × 10^5^ g/mol. Polypropylene carbonate (PPC, Nanyang Zhongju Tianguan Co., Ltd., Nanyang, China) exhibited an MFR of 12. 7 g·10 min^−1^ (190 °C, 2.16 kg) and molecular weight characteristics of Mw = 5.87 × 10^4^ g/mol, Mw/Mn = 3.65.

### 2.2. Preparation of the Biodegradable Composite Films

Prior to extrusion and pelletization, PBAT, PLA, and PPC were dried in a vacuum oven at 60, 80, and 50 °C, respectively, for 12 h to remove moisture. The dried PBAT, PLA, and PPC were then melt-blended at different compositions and pelletized. The PBAT/PLA mass ratio was fixed at 90/10, while PPC was added at mass fractions of 0%, 5%, 10%, 15%, and 20%, based on the total mass of the PBAT/PLA/PPC blend. The mixtures were extruded and pelletized using a twin-screw extruder (LJPS20-PLC, D = 20 mm, L/D = 50, Lianjiang Machinery Co., Ltd., Suzhou, China) over a temperature range of 160–180 °C. The resulting pellets were subsequently processed into films with a single-screw extrusion film blowing machine (SCM25-PLC, D = 25 mm, L/D = 28, Lianjiang Machinery Co., Ltd.) at 180 °C and a screw speed of 15 rpm. The biaxially oriented films were produced with a blow-up ratio of 3:1, and had an average thickness of 30 ± 5 μm. The resulting samples were designated PPC-0, 5%, 10%, 15%, 20%, respectively. The details of the preparation were given in Table 1.

### 2.3. Characterizations

#### 2.3.1. Rheological Properties

Rheological measurements were performed using a stress-controlled rheometer (Anton Paar, Graz, Austria) in dynamic oscillatory mode at 180 °C. The strain was fixed at 0.1%, and the angular frequency was swept from 0.01 to 100 rad·s^−1^.

#### 2.3.2. Scanning Electron Microscopy (SEM)

The surface and cross-sectional morphology of the mulch films was examined by scanning electron microscopy (ZEISS Sigma 360, Oberkochen, Germany). Film samples were fractured in liquid nitrogen, and mounted on specimen stubs using conductive adhesive tape and sputter-coated with a 10 nm layer of gold using an ion sputter coater. The observations were conducted at an accelerating voltage of 3 kV.

#### 2.3.3. X-Ray Diffraction (XRD) Test

X-ray diffraction measurements were conducted in continuous scanning mode using Cu Kα radiation and a Ni filter with X-ray diffractometer (Bruker, Kasruhe, Germany). The tube voltage and current were set at 30 kV and 10 mA, respectively, with a wavelength (λ) of 0.154 nm. The scanning range was 10.0–60.0°, with a step size of 0.02°.

#### 2.3.4. X-Ray Photoelectron Spectroscopy (XPS)

X-ray photoelectron spectroscopy was used to analyze the surface elemental composition of the different samples by X-ray photoelectron spectrometer (Thermo Fisher Scientific K-Alpha, Waltham, MA, USA), with Al Kα radiation as the excitation source. Survey spectra were collected at a pass energy of 150 eV with a step size of 1 eV, while high-resolution spectra were acquired at a pass energy of 50 eV with a step size of 0.1 eV.

#### 2.3.5. Differential Scanning Calorimetry (DSC)

Differential scanning calorimetry (DSC, Q20, TA, New Castle, DE, USA) was performed under a nitrogen atmosphere at a nitrogen flow rate of 50 mL·min^−1^. During the first heating cycle, the samples were heated from 20 to 200 °C and held at 200 °C for 2 min to remove adsorbed moisture. The samples were then cooled from 200 to −50 °C to examine the crystallization process of the films, followed by reheating to 200 °C. Both heating and cooling were conducted at a rate of 10 °C/min.

#### 2.3.6. Mechanical Properties

The mechanical properties of the mulch films in the machine direction (MD) and transverse direction (TD) were measured using a universal testing machine (HY-0580, Shanghai Hengyi Testing Instruments Co., Ltd., Shanghai, China) with reference to GB/T 35795-2017 [40]. The testing speed was 200 mm·min^−1^. Before testing, the film samples were cut into rectangular strips measuring 1 × 10 cm. For each composition, at least five independent specimens were tested, and the results are reported as the mean ± standard deviation (SD).

#### 2.3.7. Optical Properties

The light transmittance and haze of the composite films were measured using a transmittance/haze meter (Shanghai Precision Scientific Instrument Co., Ltd., Shanghai, China) in accordance with GB/T 2410-2008 [41]. Determination of the Luminous Transmittance and Haze of Transparent Plastics. A UV–Vis spectrophotometer (UV-3600 Plus, Shimadzu, Kyoto, Japan) was used to determine the spectral transmittance of the films over the wavelength range of 200–800 nm.

#### 2.3.8. Water Vapor Barrier Properties

The water vapor barrier properties were evaluated by calculating the water vapor permeability (WVP) with a water vapor transmission rate (WVTR) tester (W3/010, PERME, Jinan Labthink Electromechanical Technology Co., Ltd., Jinan, China) with the following equation:

$$WVP=\frac{WVTR\cdot d}{P\cdot \left(RH_{1}-RH_{2}\right)}$$

where d is the film thickness (μm), P is the saturated water vapor pressure (0.0066 MPa at 38 °C), and RH_1_ and RH_2_ are the relative humidities on the two sides of the film, respectively.

#### 2.3.9. Thermogravimetric (TG) Analysis

Thermogravimetric analysis was performed under an inert nitrogen (N_2_) atmosphere over a temperature range of 25–800 °C at a heating rate of 10 °C·min^−1^ (HITACHI, Tokyo, Japan).

## 3. Results

### 3.1. Effects of PPC Content on the Chemical and Crystalline Structures of the Blends

#### 3.1.1. XPS Analysis of the Composite Mulch Films

**Figure 1:**
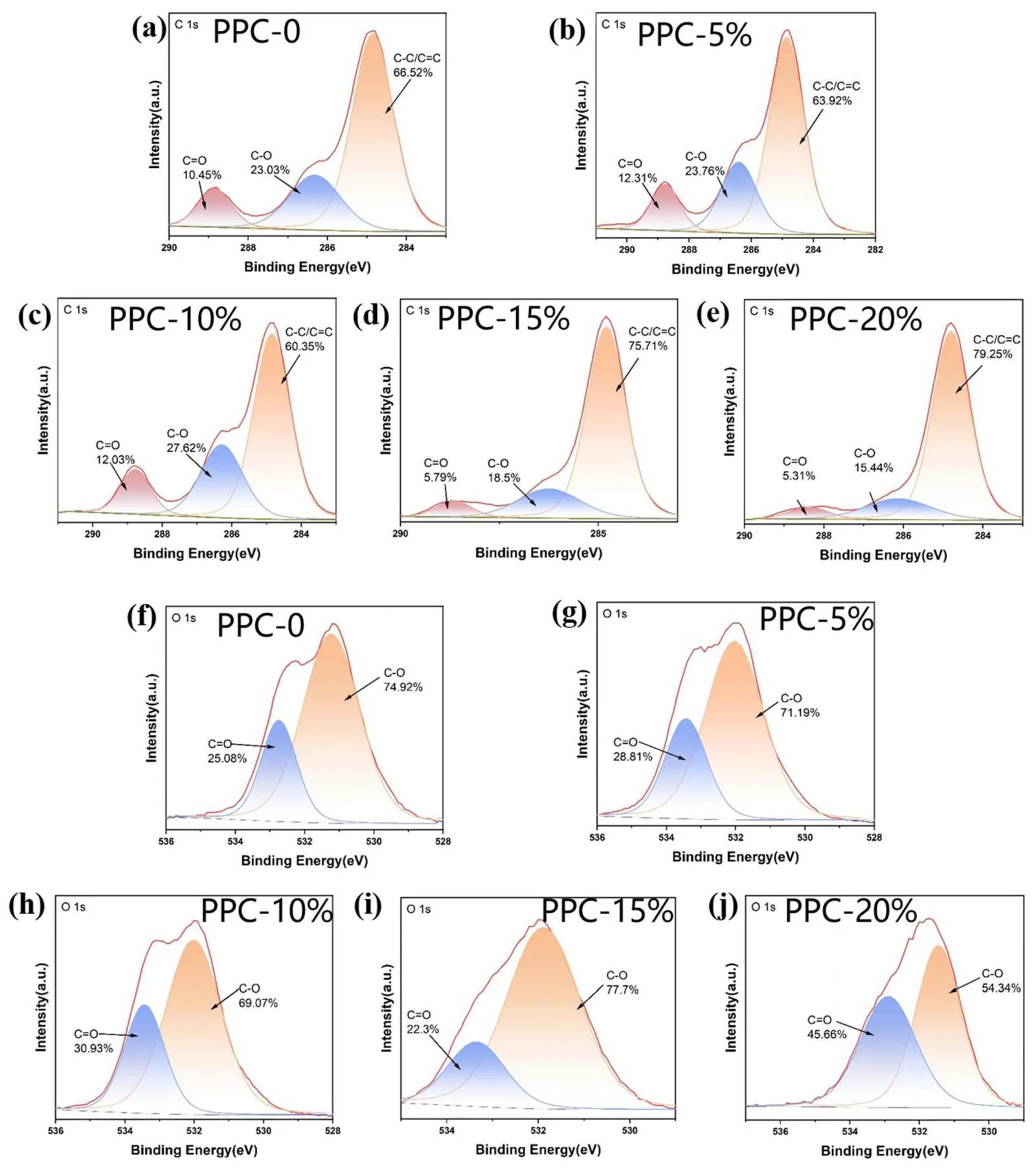
XPS of C 1s (**a**–**e**) and O 1s (**f**–**j**) at different PPC contents.

To clarify how PPC content affects the surface chemical states and component distribution of the ternary blends, XPS was used to analyze the chemical states of elements on the film surface. The high-resolution spectra of carbon and oxygen at different PPC contents were calibrated and peak-fitted [42], and the results are presented in Figure 1. Figure 1a–e showed the high-resolution C 1s spectra. Each spectrum was deconvoluted into three peaks with binding energies of (284.8 eV ± 0.2) eV, (286.5 ± 0.2) eV, and (289.0 ± 0.2) eV, corresponding to C-C/C=C, C-O, and C=O, respectively [18]. As the PPC content increased from 0% to 10%, the relative content of C-C/C=C decreased steadily from 66.52% to 60.35%, whereas that of C-O increased markedly from 23.03% to 27.62%. A slight increase was also observed in the proportion of C=O. This trend suggests good interfacial compatibility between PPC and the PBAT/PLA matrix when the PPC content is ≤10% [43]. PPC contains abundant carbonate groups along its molecular chains, and its incorporation therefore increases the density of oxygen-containing polar functional groups on the film surface, resulting in higher surface polarity [44]. When the PPC content reached 15%, however, the relative proportions of the fitted peaks changed sharply. The C-C/C=C content increased abruptly to 75.71%, while the proportions of C-O and C=O both decreased substantially. This trend became even more pronounced at a PPC content of 20%, where C-C/C=C reached 79.25%. The abrupt change indicates that the blend crosses a critical threshold of thermodynamic compatibility at approximately 15%, resulting in pronounced phase separation [45]. Because of differences in surface energy among the components, the PPC phase may migrate toward the interior of the film or become encapsulated by the continuous PLA/PBAT phase. Consequently, the surface layer becomes dominated by the PBAT component, which is rich in aromatic-ring C-C/C=C structures. This leads to a sharp increase in the hydrocarbon fraction at the surface and a substantial reduction in oxygen-containing functional groups [46]. Figure 1f–j presented the high-resolution O 1s spectra, which were deconvoluted into two peaks at binding energies of (532.0 eV ± 0.2) eV and (533.5 ± 0.2) eV, assigned to C=O and C-O, respectively. The peak-fitting results for oxygen are consistent with those obtained for carbon. As the PPC content increased from 0% to 10%, the relative proportion of C=O rose from 25.08% to 30.93%, accompanied by a corresponding decrease in C-O, further confirming the effective enrichment of PPC at the film surface. This is because the PPC molecule chain contains abundant carbonate groups, which significantly increase the proportion of surface carbonyl groups [47]. At 15% PPC, the proportion of C=O dropped sharply to 22.3%, whereas C-O increased to 77.7%, again confirming phase separation in the surface layer and indicating that the surface was reoccupied by the PBAT/PLA matrix with a higher C-O ratio. Notably, an unusual reversal was observed when the PPC content reached 20%. The C=O fraction increased to 45.06%, while C-O decreased to 54.34%, approaching the theoretical C-O/C=O stoichiometric ratio of approximately 1:1 in PBAT/PLA [48]. This behavior indicates substantial morphological reconstruction of the film surface at the high PPC content. Although the carbon spectra show that the surface remains dominated by the carbon framework of PBAT, the relative distribution of the remaining oxygen-containing species tends to return toward its expected composition. It is therefore inferred that a high PPC content induces a new multiphase microstructural arrangement within the PBAT matrix [49]. Taken together, the XPS results indicate that the ternary blend has a critical compatibility threshold between 10% and 15% PPC. At PPC contents of ≤10%, PPC is effectively enriched at the surface and improves interfacial interactions. Once the PPC content reaches 15% or higher, pronounced phase separation occurs and the surface chemical composition changes markedly, followed by a secondary reconstruction of the surface morphology at 20% PPC.

#### 3.1.2. XRD Analysis of the Composite Mulch Films

**Figure 2:**
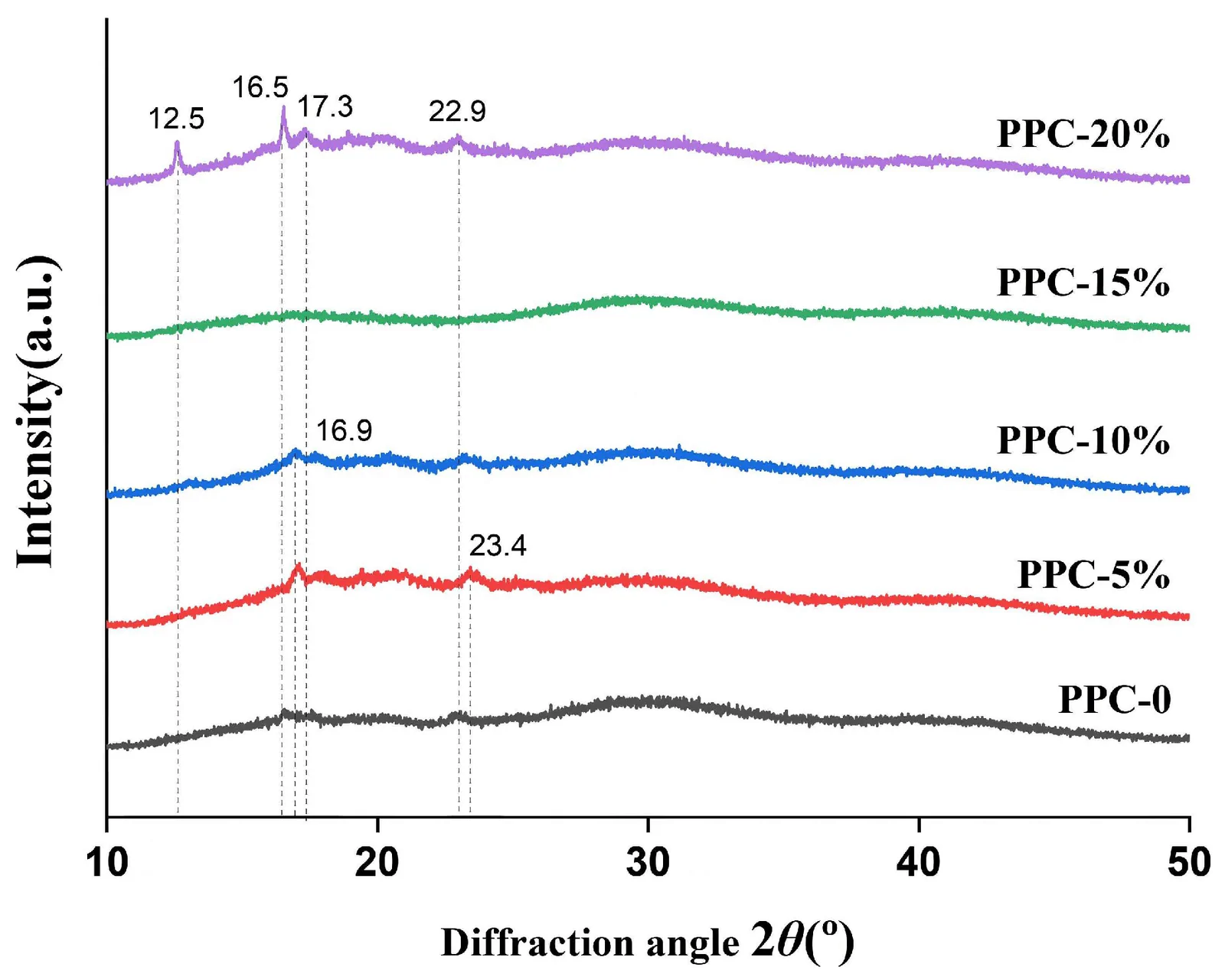
XRD patterns of composite mulch films with different PPC contents.

To determine the effect of PPC content on the bulk condensed-state structure of the blends, the crystalline structures of the composite mulch films were analyzed by XRD, and the resulting diffraction patterns are shown in Figure 2. At relatively low PPC contents (0–15%), the XRD patterns exhibit pronounced amorphous characteristics. The PPC-0, PPC-5%, PPC-10%, and PPC-15% samples all show a broad diffuse halo centered at approximately 2θ = 20°, with no sharp crystalline diffraction peaks observed [50]. These results indicate that when the PPC content does not exceed 15%, the PBAT/PLA/PPC ternary blend remains highly disordered and amorphous throughout the bulk phase. This behavior can be attributed to the good dispersion of PPC molecular chains within the matrix and their extensive chain entanglement with PBAT/PLA [29]. Such physical interlocking effectively restricts the ordered arrangement and regular packing of PBAT and PLA chain segments, thereby suppressing their crystallization. When the PPC content increases to 20%, however, the diffraction pattern changes markedly, with sharp peaks emerging at 2θ = 12.5°, 16.5°, 17.3°, and 22.9° [51,52,53]. This change indicates the formation of crystalline domains with more perfect lattice structures and larger crystallite sizes within the PBAT/PLA matrix [54]. As PPC aggregated into separate domains, the originally constrained PBAT/PLA molecular chains in certain localized regions were released. Under the extensional flow field during the blown-film process, these released chain segments underwent strain-induced orientation and packing, leading to the formation of crystallites with more perfect lattice structures [29]. The appearance of sharp XRD peaks thus reflects an improvement in the crystalline lattice order and crystallite size rather than a significant increase in the overall bulk crystallinity. Overall, the results indicate the presence of a critical PPC-content threshold. Below 15%, the system maintains a fully amorphous and relatively homogeneous structure. Once this threshold is exceeded, phase separation in the bulk phase induces the crystallization of PBAT/PLA. This observation is highly consistent with the XPS results.

### 3.2. Effect of PPC Content on the Microstructure and Interfacial Compatibility of the Blends

#### 3.2.1. Microstructure of the Composite Mulch Films

**Figure 3:**
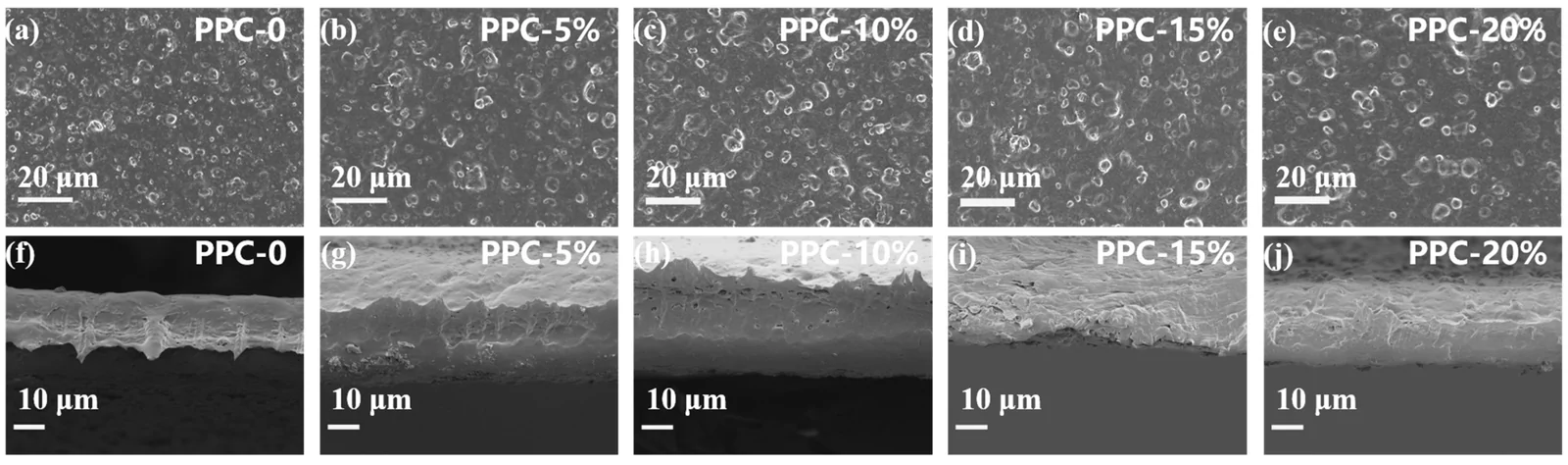
Surface SEM images (**a**–**e**) and cross-sectional SEM images (**f**–**j**) of composite mulch films with PPC contents of 0, 5%, 10%, 15% and 20%.

To directly examine the effects of PPC content on the microstructure and interfacial compatibility of the blends, the surface (Figure 3a–e) and cross-sectional (Figure 3f–j) morphologies of the composite mulch films were observed by SEM, as shown in Figure 3. Figure 3a–c show that at PPC contents of 0–10%, the film surfaces are generally smooth and uniform, with only a small number of micron-sized bright circular regions. These features are likely dispersed-phase microdomains formed as a result of differences in surface tension among the blend components [28]. The relatively uniform morphology indicates good interfacial compatibility between PPC and the PBAT/PLA matrix, allowing the components to form a comparatively homogeneous blend. This deduction is further corroborated in Figure 3f–h, where the fracture surfaces are relatively smooth and flat, with no visible phase boundaries or holes, indicating strong interfacial adhesion and a uniform internal structure. When the PPC content increases to 15% (Figure 3d), the surface morphology begins to deteriorate noticeably. The initially small bright circular domains become larger and more numerous, while the film surface develops more pronounced irregularities. These changes suggest the onset of microscopic phase separation within the blend [28]. Correspondingly, the cross-sectional image in Figure 3i presents a notably rougher fracture surface with emerging micro-voids and irregular fracture patterns, further confirming the onset of internal phase separation. At a PPC content of 20% (Figure 3e), a large ring-shaped protrusion is clearly visible, accompanied by the highest surface roughness among the samples. Such a pronounced structural defect provides direct physical evidence of macroscopic phase separation. This may occur because the excessive PPC can no longer be effectively encapsulated and stably accommodated by the continuous PBAT/PLA phase. As a result, PPC forms large, separate aggregates, accompanied by severe interfacial stress-induced debonding [55]. This macroscopic phase separation is explicitly reflected in Figure 3j, which displays a highly rough and fragmented fracture surface with large cavities and obvious interfacial debonding, directly evidencing the structural failure and poor compatibility induced by the excessive PPC content.

#### 3.2.2. Rheological Properties of the Composite Mulch Films

**Figure 4:**
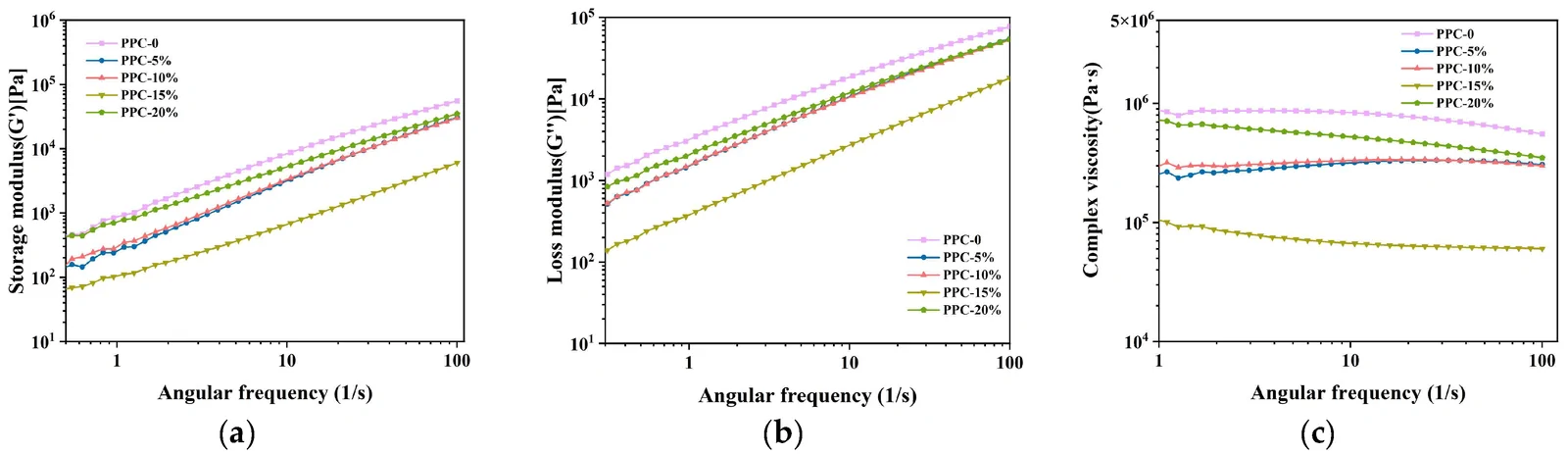
Dynamic viscoelastic curves of composite mulch films with different PPC contents: (**a**) storage modulus; (**b**) loss modulus; (**c**) complex viscosity.

Melt rheological behavior provides important insight into the microstructure, intercomponent interactions, and processing characteristics of polymer blends [18]. To investigate the effects of PPC content on molecular-chain entanglement and interfacial interactions in the molten ternary blends, dynamic frequency sweep tests were performed on the composite pellets at 180 °C using a rotational rheometer. The resulting curves of storage modulus (G′), loss modulus (G″), and complex viscosity (η*) as a function of angular frequency (ω) are shown in Figure 4. Overall, G′, η* and G″ increased with increasing angular frequency for all samples, while the complex viscosity exhibited typical shear-thinning behavior over the entire frequency range. Nevertheless, substantial differences were observed in the absolute values of the moduli and viscosity among samples with different PPC contents, with an overall decrease followed by an increase as the composition changed. As shown in Figure 4a,b, the PPC-free matrix exhibited the highest G′ and G″ values throughout the frequency range, indicating the greatest melt rigidity and strongest elastic response [31]. With the addition of 5% and 10% PPC, the modulus curves shifted progressively downward, although the decreases were relatively small. This suggests that the incorporation of a small amount of PPC reduces the density of molecular-chain entanglements to some extent without disrupting the overall network structure of the blend. When the PPC content reached 15%, however, both G′ and G″ decreased sharply and remained at the lowest levels throughout the frequency range tested, indicating a pronounced loss of melt strength. In conjunction with the XRD and SEM results described above, this behavior is likely attributable to insufficient adhesion between the dispersed and continuous phases under the melt shear field. Relative slippage at the interface consequently occurs, sharply reducing the overall resistance of the melt to deformation [34]. At 20% PPC, the moduli did not decrease further but instead showed a substantial recovery. Both the G′ and G″ curves were considerably higher than those of the samples containing 5% and 10% PPC and even approached the values of the neat matrix. This recovery reflects the enhanced interfacial elasticity associated with the formation of a droplet-matrix morphology, which can be attributed to the morphological transition induced by macroscopic phase separation [56].

Figure 4c presented the complex viscosity (η*) of all samples as a function of angular frequency. With increasing PPC content, η* followed a non-monotonic trend. At 5% and 10% PPC, η* remained nearly constant across the frequency range, exhibiting an almost Newtonian plateau, which suggested that PPC chains effectively disrupt the PBAT/PLA entanglement network and act as a lubricating diluent [31]. At 15% PPC, η* dropped dramatically to the lowest values, which provided strong quantitative evidence for severe phase separation and interfacial debilitation at this critical composition. At a PPC content of 20%, a marked increase in η* was observed, which was consistent with the variations in G′ and G″. This was attributed to the transition of the composite system from a droplet-matrix structure to a more complex heterogeneous morphology.

### 3.3. Effect of PPC Content on the Thermal Properties of the Composite Films

#### 3.3.1. DSC Analysis of the Composite Mulch Films

**Figure 5:**
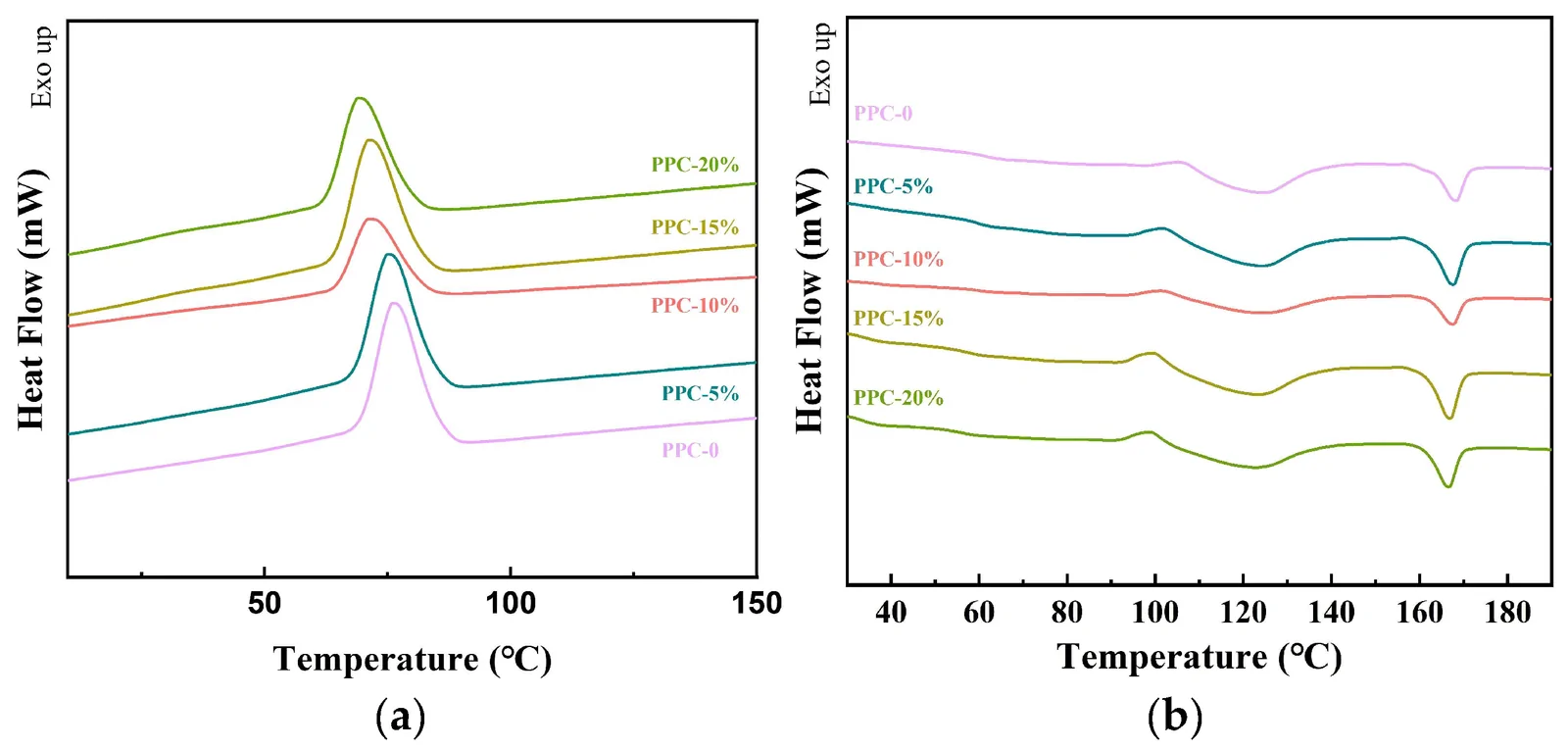
DSC curves of composite mulch films with different PPC contents: (**a**) first cooling cycle; (**b**) second heating cycle.

The thermal transition behavior of polymer blends directly reflects intercomponent compatibility, molecular-chain mobility, and changes in the condensed-state structure [18]. To further investigate the effects of PPC content on the non-isothermal crystallization behavior and melting characteristics of the ternary system, DSC analysis was performed on the composite mulch films. The DSC curves are presented in Figure 5, and the corresponding thermal parameters are summarized in Table 2. As shown in Figure 5a, during the first cooling cycle, the crystallization temperature (T_c_) decreased continuously from 76.27 °C to 69.16 °C with increasing PPC content, while the crystallization enthalpy (ΔH_c_) also progressively decreased, reaching 10.73 J/g. At PPC contents up to 10%, this suppression of crystallization is attributed to the well-dispersed PPC chains, which physically intertwined with PBAT/PLA segments and hindered their regular chain folding and nucleation during cooling [37]. The second heating curves (Figure 5b) show that the glass transition temperature (T_g_) initially decreased and then increased with increasing PPC content. When the PPC content increased to 15% and 20%, although Tc and ΔH_c_ continue to decrease, Tg increased again to values close to that of the neat matrix. This trend suggests that phase-separated interfaces and PPC-rich regions physically impede the long-range migration and diffusion of PBAT/PLA chains during non-isothermal cooling [28]. Consequently, the overall crystallization enthalpy is reduced. This finding is consistent with the characterization results discussed above.

#### 3.3.2. Thermogravimetric Analysis of the Composite Mulch Films

**Figure 6:**
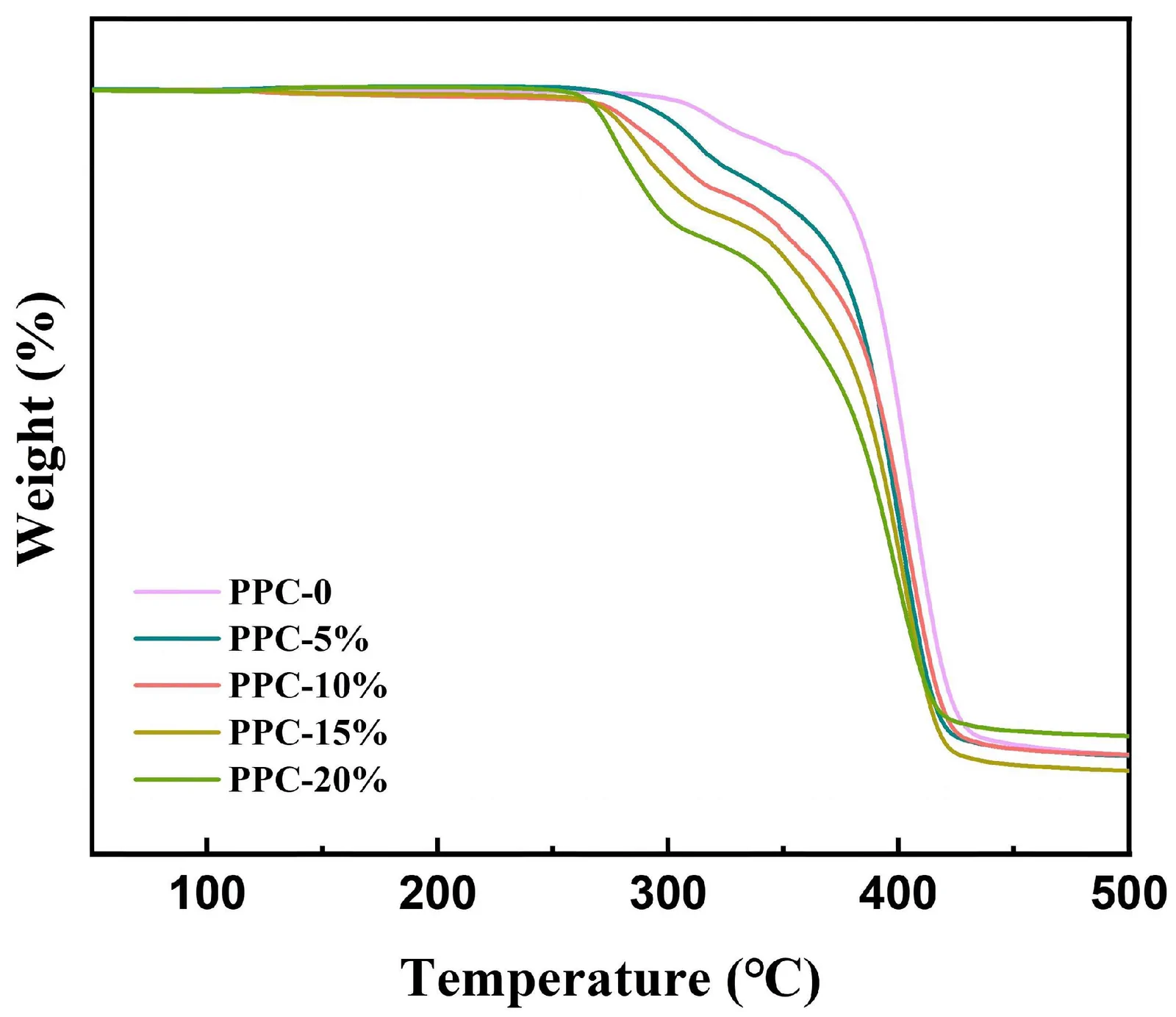
TG curves of composite mulch films with different PPC contents.

The thermal stability of polymer blends is closely related to the weather resistance and service life of mulch films during both processing and field use [18]. To examine the effect of PPC content on the thermal degradation behavior of the ternary system, programmed thermogravimetric measurements were performed on the composite films under a nitrogen atmosphere. The resulting TG curves are shown in Figure 6. All samples exhibit the typical single-stage degradation behavior of polymers. However, clear and systematic differences are observed in the onset of decomposition and the amount of char residue at high temperatures as the PPC content changes. The TG curves begin to diverge noticeably at approximately 250–280 °C. With increasing PPC content, the curves progressively shift toward lower temperatures. The neat matrix exhibits the highest thermal stability, whereas the onset of decomposition for PPC-15% and PPC-20% occurs approximately 30–40 °C earlier than that for PPC-0, with appreciable mass loss beginning at lower temperatures. These results clearly indicate that the incorporation of PPC reduces the overall thermal stability of the blend, particularly at PPC contents of 15% and above. With respect to the char residue at high temperatures, PPC-20% shows a slightly higher final char yield than the other samples. This difference appears to be associated with the phase-separation-induced crystallization observed in the XRD analysis. The molecular chains within the crystalline regions are packed more closely and regularly and may therefore be more likely to undergo crosslinking and carbonization during high-temperature pyrolysis, leaving a small amount of thermally stable carbonaceous residue [57]. Overall, the incorporation of PPC inevitably compromises the thermal stability of the composite films to some extent. When the PPC content is maintained at or below 10%, the system retains an acceptable thermal-processing window while maintaining good compatibility. Once the PPC content reaches 15% or higher, however, the deterioration in thermal stability associated with phase separation substantially reduces the heat resistance of the films and may increase the risk of premature aging during processing and prolonged exposure to high temperatures under field conditions. As shown in Table 3, the temperatures at 5% and 10% weight loss (T_5%_ and T_10%_), and maximum decomposition temperature (T_max_) decreased progressively with increasing PPC content. The addition of only 5% PPC reduced T_5%_ from 322.81 °C to 306.82 °C, and a further increase to 10% PPC resulted in a more substantial drop to 283.45 °C, indicating that the incorporation of PPC accelerates the onset of thermal decomposition. The T_max_ values followed a similar decreasing trend, falling from 401.23 °C (PPC-0) to 385.00 °C (PPC-20%). The reduction in thermal stability became particularly pronounced at PPC contents of 15% and above, which is consistent with the phase separation behavior discussed in Section 3.1 and Section 3.2.

### 3.4. Effect of PPC Content on the Mechanical Properties of the Composite Mulch Films

**Figure 7:**
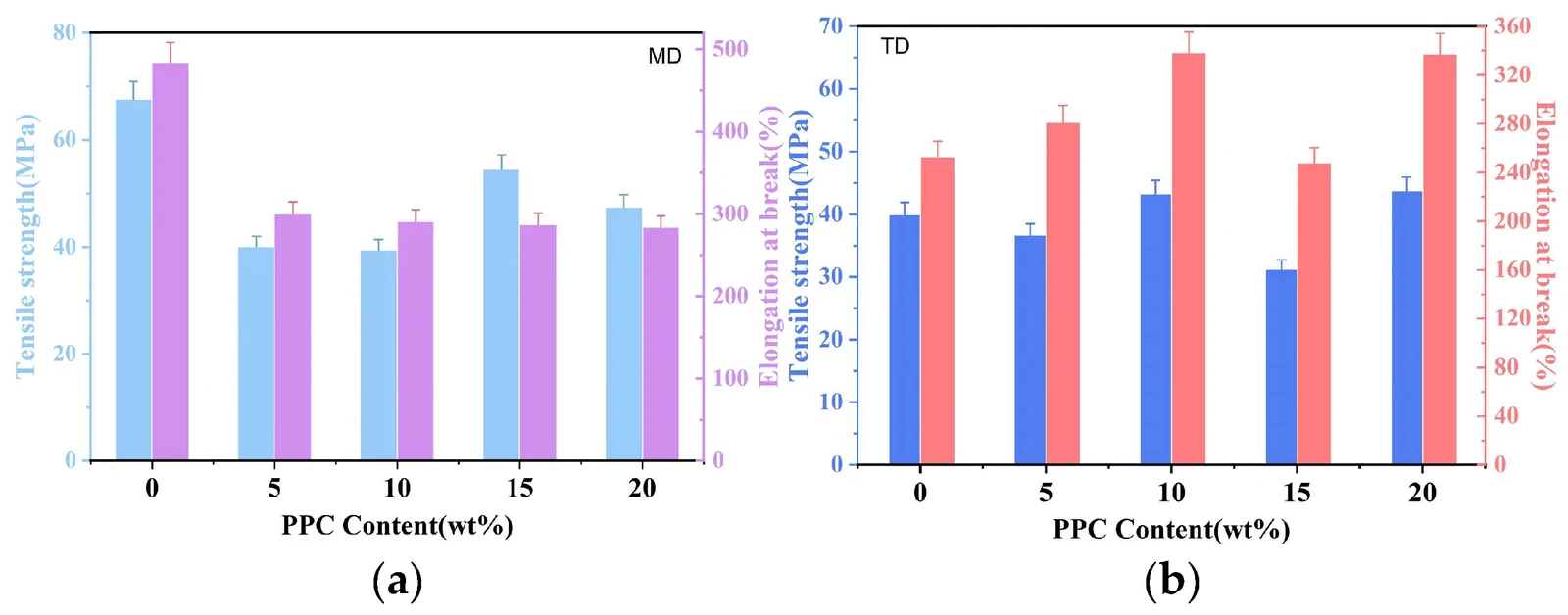
Tensile strength and elongation at break of composite mulch films with different PPC contents: (**a**) MD; (**b**) TD.

Figure 7 presents the mechanical properties of the composite mulch films in the machine direction (MD) and transverse direction (TD) at different PPC contents. As shown in Figure 7a, the tensile strength in the MD first decreases, then increases, and subsequently decreases again with increasing PPC content. The neat matrix exhibits the highest tensile strength of 67.5 ± 3.37 MPa. At PPC contents of 5% and 10%, the tensile strength is approximately 40 MPa, increasing to about 54.5 ± 2.72 MPa at 15% before decreasing again to 47.4 ± 2.37 MPa at 20%. Upon addition of 5% PPC, the elongation at break in the MD decreased from 484 ± 24.2% to 299.67 ± 14.98%. With further increasing PPC content, the elongation at break remained nearly constant in the range of 283~290%. This is because the molecular chains in blown films are highly oriented along the MD, and the disruption of the PBAT/PLA continuous phase by PPC leads to an irreversible reduction in the MD elongation [58]. As shown in Figure 7b, the tensile strength in the TD varies more markedly with PPC content, fluctuating between 31 and 44 MPa and reaching its lowest value of 31.18 ± 1.56 MPa at 15% PPC. The introduction of PPC significantly improved the elongation at break of the composite films in the TD. Specifically, at PPC contents of 10% and 20%, the elongation at break increased by 33.72% and 33.33%, respectively, compared with that of the neat matrix. Notably, at 15% PPC, the elongation at break dropped to 248%, approaching the value of the neat matrix. This decrease is presumably attributable to severe phase separation and interfacial slip occurring in the composite system containing 15% PPC [59]. The composite film containing 10% PPC exhibits the best transverse mechanical properties, with a tensile strength of 43.25 ± 2.16 MPa and an elongation at break of 338.33 ± 16.92%, both exceeding those of the neat matrix. These variations in mechanical properties can be interpreted in terms of the evolution of the blend microstructure. At PPC contents of 5–10%, PPC shows good compatibility with the matrix, and the optimum transverse mechanical properties are obtained at 10% PPC [36]. However, the incorporation of PPC disrupts the preferential orientation of PBAT/PLA in the MD, leading to a reduction in longitudinal mechanical performance [60]. This performance deterioration can be explained by two competing mechanisms. PPC is an amorphous and flexible aliphatic polycarbonate. Its intrinsic tensile strength is much lower than that of PBAT and PLA [34]. When introduced into the rigid PBAT/PLA matrix, PPC inevitably reduces the volume fraction of the load-bearing rigid phase. This consequently lowers the overall tensile modulus and strength of the composite. In blown film extrusion, the high tensile strength in the MD primarily originates from strain-induced orientation and alignment of PBAT/PLA chains along the stretching direction [61]. When well-dispersed PPC chains are present, they physically entangle with PBAT/PLA segments. This increases the interchain entanglement density. Such enhanced entanglement restricts the relaxation and reorientation of PBAT/PLA chains during cooling. It effectively weakens the molecular orientation along the MD. As a result, the material anisotropy is reduced [38]. The tensile strength along the MD decreases, while the elongation and strength in the TD are markedly improved. When the PPC content increases to 15%, severe phase separation occurs, and interfacial debonding results in a marked deterioration of the mechanical properties in both the MD and TD. With a further increase to 20%, the macroscopic phase separation caused by excess PPC releases the constraints on the PBAT/PLA matrix and induces pronounced crystallization. As a result, the transverse mechanical properties of the composite film partially recover. Taken together, the results show that PPC provides the most effective toughening at a content of 10%, indicating that 10% is the optimal PPC addition level for this ternary blend system.

### 3.5. Effect of PPC Content on the Water Vapor Barrier Properties of the Composite Mulch Films

**Figure 8:**
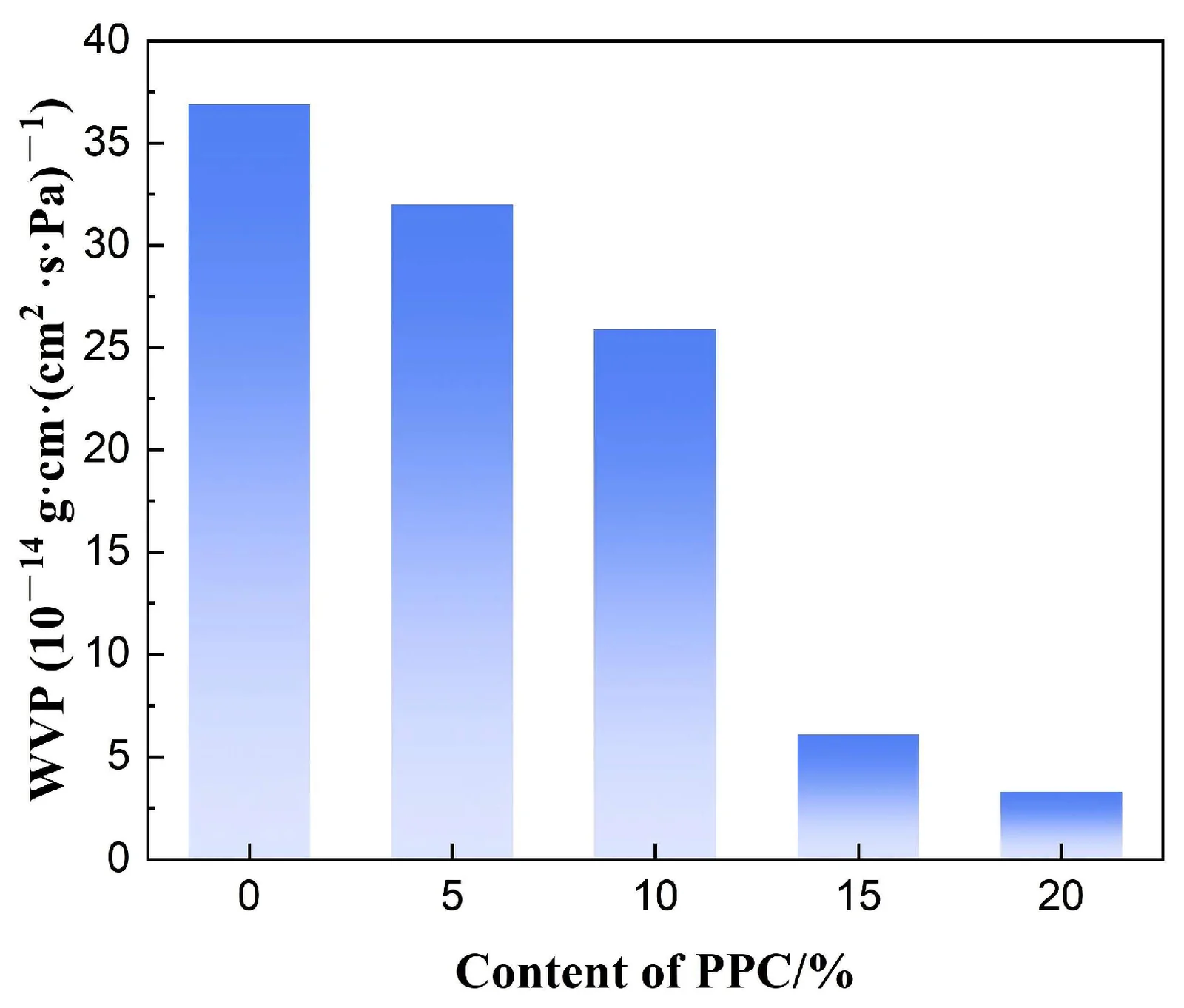
WVP coefficients of composite mulch films with different PPC contents.

The incorporation of PPC as a barrier material into the PBAT/PLA matrix improves the water vapor barrier performance of the composite mulch films [36]. Figure 8 compares the WVP coefficients of films containing different amounts of PPC. The neat matrix film exhibits the highest WVP coefficient, at 3.692 × 10^−13^ g·cm·(cm^2^·s·Pa)^−1^. As the PPC content increases, the water vapor permeability coefficient of the composite films gradually decreases. When the PPC content is 10%, the WVP coefficient decreased to 2.592 × 10^−13^ g·cm·(cm^2^·s·Pa)^−1^, with a 29.8% reduction versus the neat blend. A particularly pronounced decrease is observed as the PPC content increases from 10% to 15%, with the coefficient falling to only 6.101 × 10^−14^ g·cm·(cm^2^·s·Pa)^−1^, representing an 83.5% reduction relative to the neat matrix film. At relatively low PPC contents, PPC is uniformly dispersed throughout the PBAT/PLA matrix, increasing the tortuosity of the diffusion pathways available to water molecules and thereby producing a steady decrease in the WVP coefficient [18]. When the PPC content reaches 15% or higher, severe phase separation occurs in the composite system, as discussed above, inducing crystallization of the PBAT/PLA matrix. The resulting densely packed crystalline regions are difficult for water molecules to penetrate and therefore act as effective barriers to diffusion, leading to the pronounced reduction in WVP [36].

### 3.6. Effect of PPC Content on the Optical Properties of the Composite Mulch Films

#### 3.6.1. UV-Shielding Performance of the Composite Mulch Films

**Figure 9:**
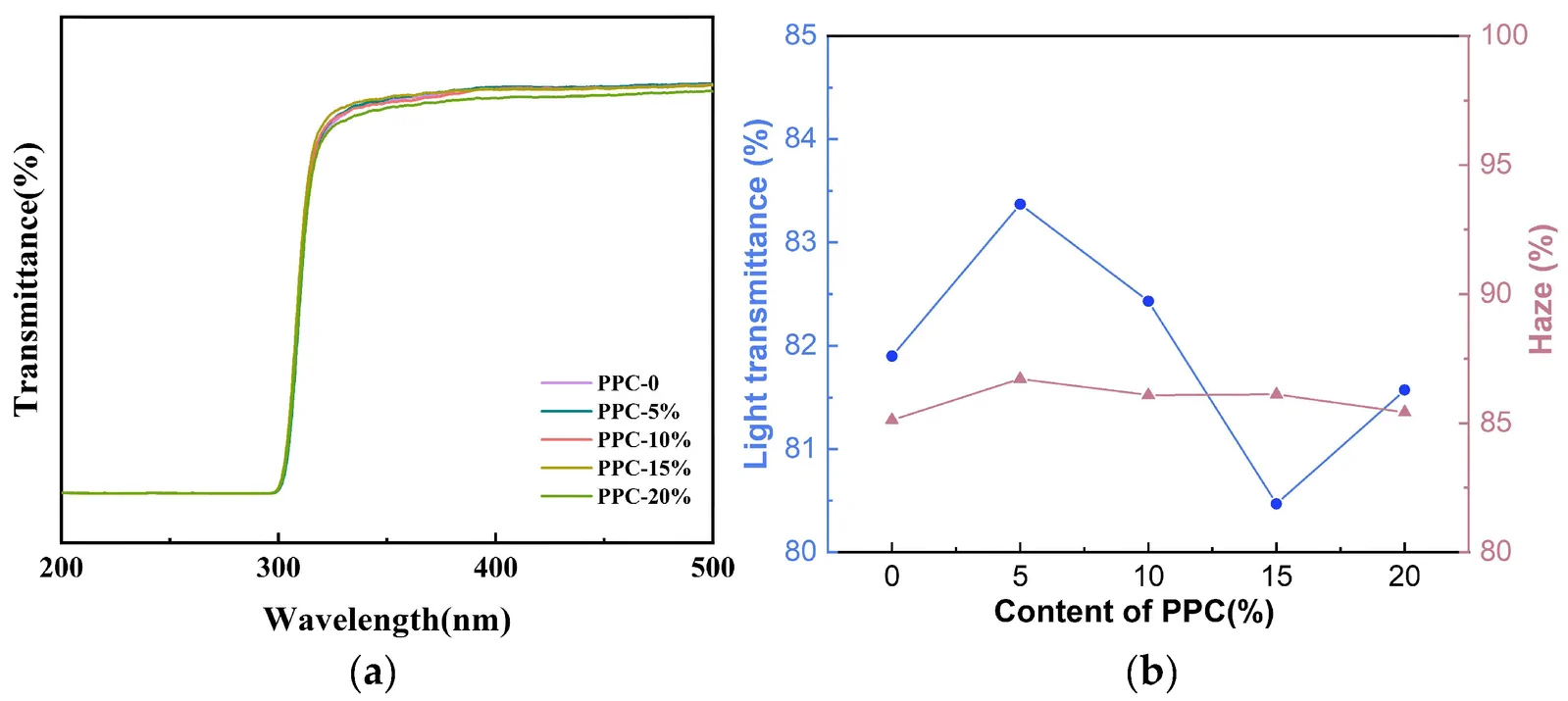
Optical properties of composite mulch films with different PPC contents: (**a**) UV–Vis spectra; (**b**) light transmittance and haze.

Figure 9a shows the UV–Vis transmission spectra of the composite mulch films with different PPC contents. As can be seen from the spectra, the UV transmittance of all composite films is close to zero over the wavelength range of 200–320 nm, regardless of PPC content. This indicates that the incorporation of PPC does not affect the UV-shielding performance of the PBAT/PLA matrix.

#### 3.6.2. Light Transmittance and Haze of the Composite Mulch Films

Figure 9b presents the light transmittance and haze of the composite mulch films with different PPC contents. When 5–10% PPC is incorporated, the films exhibit higher light transmittance than the neat matrix. This finding further supports the previous conclusion that PPC at relatively low contents has good compatibility with the matrix [18]. The resulting uniform microstructure reduces light scattering and consequently improves the light transmittance of the composite films. When the PPC content reaches 15% or higher, however, the light transmittance decreases markedly. This can be attributed to phase separation: the dimensions of the incompatible dispersed domains become comparable to the wavelength of visible light, resulting in strong Mie scattering and a sharp reduction in light transmittance [62]. Unlike light transmittance, the haze of all samples remains relatively stable at approximately 85–86%, with only minor variations. This suggests that extensive diffuse scattering occurs as light passes through the films regardless of whether phase separation is present within the matrix, resulting in little difference in haze among the samples.

## 4. Conclusions

In this work, the effects of PPC content (0–20%) on the structure and properties of PBAT/PLA/PPC ternary composite films were systematically investigated, and the critical compatibility threshold was identified in the range of 10–15 wt%. At PPC contents ≤10%, the system remained homogeneous and amorphous, with PPC effectively enriched at the surface and enhancing interfacial adhesion, thereby achieving an optimal balance of overall performance. At contents exceeding 15%, pronounced phase separation occurred, inducing crystallization of PBAT/PLA, accompanied by deteriorated mechanical properties, reduced melt strength, and lowered thermal stability. At 20%, phase separation induces PBAT/PLA crystallization, further enhancing barrier performance but reducing thermal stability and transparency. Overall, 10% PPC was determined as the optimal loading, at which the films exhibited favorable mechanical strength, flexibility, water vapor barrier, and light transmittance. This work reveals the relationships among intrinsic composition, structure and property, providing a theoretical basis for the formulation design of biodegradable mulch films. Further field weathering and biodegradation tests are required to validate their practical agricultural performance. In addition, future work could explore the synergistic effects of compatibilizers and higher PPC contents to further optimize the overall performance.

## Figures and Tables

**Table 1:** The formulations of composite films in this work.

	PBAT Contents	PLA Contents	PPC Contents
PPC-0	90%	10%	0
PPC-5%	85.5%	9.5%	5%
PPC-10%	81%	9%	10%
PPC-15%	76.5%	8.5%	15%
PPC-20%	72%	8%	20%

**Table 2:** DSC thermal parameters of composite mulch films with different PPC contents.

Sample	ΔH_m_ (J/g)	ΔH_c_ (J/g)	T_c_ (°C)	T_m_ (°C)	T_g_ (°C)
PPC-0	3.603	13.03	76.27	171.38	41.6
PPC-5%	4.127	11.75	75.18	171.03	40.56
PPC-10%	3.967	11.66	73.13	170.94	39.7
PPC-15%	3.926	11.13	71.39	171.02	42.2
PPC-20%	3.377	10.73	69.16	171.22	41.04

**Table 3:** Thermal decomposition temperatures of composite films with different PPC contents.

Sample	T_5%_ (°C)	T_10%_ (°C)	T_max_ (°C)
PPC-0	322.81	357.91	401.23
PPC-5%	306.82	323.24	397.29
PPC-10%	283.45	301.91	394.13
PPC-15%	278.63	290.54	390.10
PPC-20%	274.67	282.91	385.00

## Data Availability

The raw data supporting the conclusions of this article will be made available by the authors on request.
